# Evolution of symbiosis with resource allocation from fecundity to survival

**DOI:** 10.1007/s00114-014-1175-1

**Published:** 2014-04-18

**Authors:** Shin Fukui

**Affiliations:** 1 Center for Ecological Research, Kyoto University, 2-509-3 Hirano, Otsu, Shiga 520-2113 Japan; 2 Agro-Meteorology Division, National Institute for Agro-Environmental Sciences, 3-1-3 Kannon-dai, Tsukuba, Ibaraki 305-8604 Japan

**Keywords:** Fitness component, Evolution of mutualism, Dual lattice model

## Abstract

Symbiosis is one of the most fundamental relationships between or among organisms and includes parasitism (which has negative effects on the fitness of the interacting partner), commensalism (no effect), and mutualism (positive effects). The effects of these interactions are usually assumed to influence a single component of a species’ fitness, either survival or fecundity, even though in reality the interaction can simultaneously affect both of these components. I used a dual lattice model to investigate the process of evolution of mutualistic symbiosis in the presence of interactive effects on both survival and fecundity. I demonstrate that a positive effect on survival and a negative effect on fecundity are key to the establishment of mutualism. Furthermore, both the parasitic and the mutualistic behaviour must carry large costs for mutualism to evolve. This helps develop a new understanding of symbiosis as a function of resource allocation, in which resources are shifted from fecundity to survival. The simultaneous establishment of mutualism from parasitism never occurs in two species, but can do so in one of the species as long as the partner still behaves parasitically. This suggests that one of the altruistic behaviours in a mutualistic unit consisting of two species must originate as a parasitic behaviour.

**Electronic supplementary material:**

The online version of this article (doi:10.1007/s00114-014-1175-1) contains supplementary material, which is available to authorized users.

## Introduction

Symbiosis, one of the most fundamental relationships between or among organisms, has traditionally been divided into three categories, namely parasitism, commensalism, and mutualism. In parasitism, the symbiont species benefits at the expense of the host; in mutualism, both species benefit, and in commensalism, the relationship has no effect on the host, but the symbiont benefits in terms of fitness (Krebs 2001; Paracer and Ahmadjian 2000). An understanding of evolution of mutualism is central to understanding ecology (Doebeli and Knowlton 1998; Herre et al. 1999; de Mazancourt et al. 2005; Sachs et al. 2004; Yamamura et al. 2004). However, recent research suggests that symbiotic roles are context-dependent and/or more complex than previously thought and that in reality symbiosis cannot always be simply defined as one of these categories (Fellous and Salvaudon 2009). For example, Johnson et al. (1997) state that the association between plants and symbiotic mycorrhizae growing around their roots is located on a mutualism-parasitism continuum. In maize, mycorrhizae positively affect crop growth in ridge-tilled fields, but in no-till fields, they result in low yields (McGonigle and Miller 1996). A bacterial endosymbiont of aphids, *Serratia symbiotica*, reduces its host’s fecundity and growth rate under normal conditions, but promotes its survival after the removal of an essential obligate endosymbiont, *Buchnera* (Douglas 1998; Koga et al. 2003). There have been a number of theoretical studies focusing on the coexistence of parasites and conditional mutualists (Hoeksema and Bruna 2000; Ferriere et al. 2002; Nuismer et al. 2003; Neuhauser and Fargione 2004; Kummel and Salant 2006), but these have not examined the evolutionary aspects of these phenomena. The evolutionary history of these symbiotic relationships is in need of further investigation.

A scenario for the evolution of symbiosis, introduced by Ewald (1987) in a review of studies about host-symbiont interactions, is that mutualism can evolve from parasitism through a reduction in harmful effects to the host. This process would be coupled with a shift of transmission between hosts from horizontal to vertical. Vertical transmission would mean that the symbiont’s fitness remained high and would be detrimental to the host. The evolutionary dynamics of the decrease in virulence attending the shift from horizontal to vertical transmission is theoretically supported (Matsuda and Shimada 1993; Yamamura 1993, 1996). However, mutualism could not become established under this scenario unless the parasitic relationship is dissolved. In addition, the symbiosis might still ultimately cost the host, because the symbiont uses essential nutrients to produce it. Genkai-Kato and Yamamura (1999) demonstrated theoretically that a parasite that benefits its host by producing a beneficial waste (cost-free) product achieves mutualism even under horizontal transmission. However, the provisioning of the resource might also cost the symbiont, because the symbiont provides essential nutrients required by the symbiont itself, as in the case of the aphid-*Serratia* association (Koga et al. 2003) or the bedbug-*Wolbachia* association (Hosokawa et al. 2010). Furthermore, Hosoda et al. (2011) showed experimentally that two independent, genetically engineered auxotrophic strains of *Escherichia coli* could become a mutualistic symbiotic unit, making use of metabolites over-supplied by each other. This suggests that the establishment of mutualism requires a phenotypic change in the form of over-production of metabolites. In Ewald’s scenario, the net effect of the parasite is realised through its virulence and transmission. Research on the evolution of symbiosis has yet to examine whether a symbiont benefits its host through altruistic behaviour such as the provisioning of metabolites.

Phylogenetic studies of symbiotic microbes suggest an alternative scenario for the evolution of mutualism (Moran and Wernegreen 2000; Sachs et al. 2011). Sachs et al. (2011) visualised the phylogenetic tree of symbiotic microbes and their symbiotic role. This study suggested that mutualism does originate from parasitism in some cases, although the mutualism of *Rhizobia* with legume plants might have become established directly from free-living organisms (Sullivan et al. 1995; Young and Haukka 1996; Sachs et al. 2010). This implies that mutualism does not necessarily originate from parasitism; the original symbionts might not always have been harmful to their hosts. This evolutionary process can be considered from the viewpoint of game theory. A lattice model incorporating the Iterated Prisoner's Dilemma (IPD) game was used to investigate the evolution of cooperation in a single species (Axelrod and Hamilton 1981). This approach has subsequently been extended to a cooperation game between two species on a dual lattice, which means that one species occupies each lattice space and the two species interact with each other on a sympatric lattice point (Doebeli and Knowlton 1998; Ezoe 2009). Yamamura et al. (2004) adopted the dual lattice model to investigate the effects of the dispersal modes of interacting species on the evolution of symbiosis and concluded that restricted dispersal promotes the evolution of mutualistic symbiosis from non-mutualistic systems. These theoretical studies were novel in that they incorporated the assembling effect of mutualisms, but they did not consider the parasitic aspect.

Here, I focus on symbiosis that simultaneously involves two opposing properties, i.e., mutualism and parasitism, in contrast to Ewald’s hypothesis (1987). These symbionts simultaneously have both positive and negative effects, and their symbiotic behaviours potentially have differential influences on the two components of recipient fitness: fecundity and survivorship. For example, *Wolbachia*, which parasitises the reproductive system of *Drosophila melanogaster*, also suppresses an RNA virus infection in its host (Hedges et al. 2008; Teixeira et al. 2008). In such a case, the positive and negative effects of the symbiont might not be additive, and their effect on the evolutionary dynamics of the symbiotic relationship is not fully understood. Travis et al. (2006) examined whether mutually beneficial or competitive interactions between two species were favoured, depending on environmental conditions correlated to fecundity or mortality. They used the dual lattice model to study how a symbiont affects its partner’s fecundity and survivorship. This revealed that extreme environmental conditions favour mutualism if they reduce the partner’s fecundity and that moderate conditions favour mutualism if they increase the partner’s survival. However, those factors reflect environmental conditions rather than characteristics of the symbiont and thus have no bearing on the evolution of mutualism from parasitism.

In this study, I investigate the evolution of symbiosis using a dual lattice space, including the effects of symbiosis on the fecundity and survivorship of both interacting species. I treat the altruistic nature of the symbiont independently from parasitism, so that a mutualistic strain is nevertheless treated as parasitic. However, the net effect of the interaction between the species is zero if the parasitic (negative) and mutualistic (positive) effects on the partner are equal. I focus on a situation where mutualism never evolves in a well-mixed population, but by incorporating spatial structure, I show that mutualism can evolve from parasitism in a host-symbiont system. This can occur if the symbiont’s parasitic behaviour benefits its survival by exploiting the host’s resources while it simultaneously devotes its resources to its host’s survival. I also show that mutualism never becomes established simultaneously in two parasitic species.

## The model

I constructed a dual lattice space model in which the two species, Sp. A and Sp. B, each inhabit one lattice. Lattices exist in parallel, following previous studies (Doebeli and Knowlton 1998; Yamamura et al. 2004; Travis et al. 2006). The model includes two strains of each species, one of which is parasitic and the other mutualistic. Each cell of each lattice is therefore characterised by one of the three states as follows: empty, parasitic individual present, and mutualistic individual present. The frequencies of the three states for Sp. A are denoted as *A*_*E*_, *A*_*P*_, and *A*_*M*_ and those for Sp. B *B*_*E*_, *B*_*P*_, and *B*_*M*_. The total frequency for each species must be equal to one, as follows:

1 $$ {A}_E+{A}_P+{A}_M=1, $$

2 $$ {B}_E+{B}_P+{B}_M=1. $$

In this system, symbiotic interaction occurs interspecifically between individuals that inhabit the same spatial position on different lattices.

**Fig. 1:**
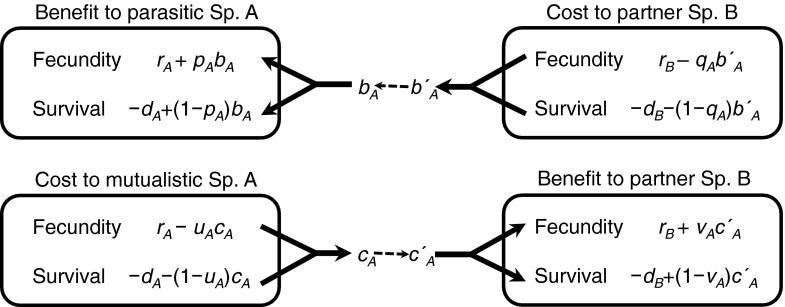
Outline of the interactions between organisms of two species, A and B, in the dual lattice model. Each organism is essentially parasitic in nature, and parasitic behaviours are represented by *b* and *b′*. Altruistic behaviours, as exhibited by mutualist strains of each organism, are represented by *c* and *c′. Arrows* represent interactions and start from the fecundity or survival of one organism and terminate in the fecundity or survival of its partner organism. *Arrows* therefore indicate the strategy for resource exploitation or allocation. The strategies, represented as binary parameters *p*, *q*, *u*, and *v*, are summarised in Table 1

Let the intrinsic reproductive and mortality rates of Sp. A be *r*_*A*_ and *d*_*A*_, and those of Sp. B be *r*_*B*_ and *d*_*B*_. Interactions between the two species in this model are based on the delivery of valuable resources. The parasitic or mutualistic behaviours of an individual influence the reproductive and mortality rates of its partner individual on the other lattice (Fig. 1). In this model, the symbiotic interaction is based on parasitism, and mutualism derives from parasitism. Mutualistic interactions in this study therefore comprise both costs and benefits (Fig. 1) to the partner species. The mutualistic relationship in this study does not necessarily increase the fitness of the partner species, relative to free-living individuals, because the partner’s fitness is considered relative to the state in which it is being parasitised.

When an individual of Sp. A is a parasite, its interaction with an individual of Sp. B takes the form of exploitation. This results in a reduction in fecundity and survival for Sp. B, denoted *q*_*A*_ *b′*_*A*_ and (1−*q*_*A*_) *b′*_*A*_, respectively, where (0 ≤ *q*_*A*_ ≤ 1). The sum of these two quantities is *b′*_*A*_. Parasitism by Sp. A enhances its fecundity and survival, denoted *p*_*A*_ *b*_*A*_ and (1−*p*_*A*_) *b*_*A*_, respectively, where (0 ≤ *p*_*A*_ ≤ 1). The sum of these quantities is *b*_*A*_. In other words, when Sp. B is a neutral host (neither parasite nor mutualist), the interaction with parasitic Sp. A results in reproductive rates of *r*_A_ + *p*_*A*_ *b*_*A*_ and *r*_B_ − *q*_*A*_ *b′*_*A*_ for Sp. A and Sp. B, respectively. Similarly, the mortality rates of Sp. A and Sp. B become *d*_A_ − (1 − *p*_*A*_ ) *b*_*A*_ and *d*_B_ + (1 − *q*_*A*_ ) *b′*_*A*_, respectively. The quantities *q*_*A*_ and *p*_*A*_ represent the relative effects of parasitism on the fecundity and survival of the parasite and recipient, respectively. The total cost to Sp. B (*b′*_*A*_) of the parasitism does not necessarily equal the total advantage to the parasite, Sp. A (*b*_*A*_).

When an individual of Sp. A is a mutualist, it interacts with an individual of Sp. B both parasitically and beneficially. The parasitic interaction is coded as above. Therefore, a mutualist devotes resources to its partner species, which results in increased fitness in the partner, relative to individuals that interact with the parasite. Sp. A reduces its fecundity and survival by *u*_*A*_ *c*_*A*_ and (1−*u*_*A*_) *c*_*A*_, respectively, where (0 ≤ *u*_*A*_ ≤ 1). The sum of these two quantities is *c*_*A*_, which is the total quantity of the resource available to Sp. B. These resources increase the fecundity and survival of Sp. B by *v*_*A*_ *c′*_*A*_ and (1−*v*_*A*_) *c′*_*A*_, respectively, where (0 ≤ *v*_*A*_ ≤ 1). When mutualistic Sp. A interacts with naïve host Sp. B, their reproductive rates become *r*_A_ + *p*_*A*_ *b*_*A*_ − *u*_*A*_ *c*_*A*_ and *r*_B_ − *q*_*A*_ *b′*_*A*_ + *v*_*A*_ *c′*_*A*_, respectively. Similarly, their mortality rates become *d*_A_ − (1 − *p*_*A*_) *b*_*A*_ + (1−*u*_*A*_) *c*_*A*_ and *d*_B_ + (1 − *q*_*A*_) *b′*_*A*_−(1−*v*_*A*_) *c′*_*A*_, respectively. A symbiotic relationship with a mutualistic strain of Sp. A is therefore effectively commensalism for Sp. B if *q*_*A*_ = *v*_*A*_ and *c′*_*A*_ = *b′*_*A*_, because the overall effect of such a mutualist on its partner is zero. Similar notation is applied to the parasitic and mutualistic strains of Sp. B, i.e., *r*_*B*_ , *d*_*B*_, *b*_*B*_, *p*_*B*_, *b′*_*B*_, *q*_*B*_, *c*_*B*_, *u*_*B*_, *c′*_*B*_, and *v*_*B*_ (Table. 1).

**Table 1:** Parameters used in the dual lattice model. Sp. X is the active symbiotic species and Sp. Y is the recipient species (*X*, *Y* ∈ *A*, *B*). Sp. X_M_ represents the mutualistic strain of Sp. X

Parameter	Description
*r*_X_	Basic reproductive rate of Sp. X
*d*_X_	Basic mortality rate of Sp. X
*b*_X_	Benefit to Sp. X of parasitically exploiting Sp. Y
*c*_X_	Cost to Sp. X_M_ of providing resources to Sp. Y
*p*_X_	Sp. X’s allocation strategy for resources parasitised from Sp. Y (Sp. X allocates 100 % of the resources to fecundity if *p*_X_ = 1, to survival if *p*_X_ = 0.)
*q*_X_	Sp. X’s parasitic behaviour strategy (100 % of the resources Sp. X parasitises affect Sp. Y’s fecundity if *q*_X_ = 1, and survival if *q*_X_ = 0.)
*u*_X_	Sp. X_M_’s resource donation strategy (Of the resources that Sp. X_M_ donates to Sp. Y, 100 % affect Sp. X_M_’s fecundity if *u*_X_ = 1, and 100 % affect its survival if *u*_X_ = 0.)
*v*_X_	Sp. X_M_’s resource allocation strategy (Of the resources that Sp. X_M_ donates to Sp. Y, 100 % affect Sp. Y’s fecundity if *v*_X_ = 1, and 100 % affect its survival if *v*_X_ = 0.)

## Unlimited dispersal

First, consider the case of no dispersal limitation for either species. When the dispersal of offspring is unlimited and random, interactions between parasitic and mutualistic strains of the two species are expected to occur with probabilities proportional to their abundance in the population. Accordingly, the frequency dynamics of *A*_*P*_ and *A*_*M*_ can be represented by the following differential equations:

3 $$ \frac{d{A}_p}{ dt}={r}_A{B}_E{A}_P{A}_E+{R}_{PP}{B}_P{A}_P{A}_E+{R}_{MP}{B}_M{A}_P{A}_E-{d}_A{B}_E{A}_P-{D}_{PP}{B}_P{A}_P-{D}_{MP}{B}_M{A}_P, $$

and

4 $$ \frac{d{A}_M}{ dt}={r}_A{B}_E{A}_M{A}_E+{R}_{PM}{B}_P{A}_M{A}_E+{R}_{MM}{B}_M{A}_M{A}_E-{d}_A{B}_E{A}_M-{D}_{PM}{B}_P{A}_M-{D}_{MM}{B}_M{A}_M, $$

where

5 $$ {R}_{PP}={r}_X+{p}_X{b}_X-{q}_Yb{\prime}_Y $$

6 $$ {R}_{MP}={R}_{PP}+{u}_Yc{\prime}_Y $$

7 $$ {R}_{PM}={R}_{PP}-{u}_X{c}_X $$

8 $$ {R}_{MM}={R}_{PP}-{u}_X{c}_X+{u}_Yc{\prime}_Y $$

9 $$ {D}_{PP}={d}_X-\left(1-{p}_X\right){b}_X+\left(1-{q}_Y\right)b{\prime}_Y $$

10 $$ {D}_{MP}={D}_{PP}-\left(1-{u}_Y\right)c{\prime}_Y $$

11 $$ {D}_{PM}={D}_{PP}+\left(1-{u}_X\right){c}_X $$

and

12 $$ {D}_{MM}={D}_{PP}+\left(1-{u}_X\right){c}_X-\left(1-{u}_Y\right)c{\prime}_Y. $$

The subscript *X* represents the symbiotic actor, and *Y* represents the recipient (*X*, *Y* ∈ *A*, *B*). In reproductive and mortality rates (e.g., *R*_*PM*_, *D*_*PP*_), the first subscript represents the symbiotic recipient, and the second represents the actor. The corresponding equations for *B*_*P*_ and *B*_*M*_ can be formulated in a similar manner.

From Eqs. (3, 4, 5, 6, 7, 8, 9, 10, 11 and 12), we get the following:

13 $$ \frac{1}{A_M}\frac{d{A}_M}{ dt}-\frac{1}{A_P}\frac{d{A}_P}{ dt}=-{c}_A\left\{{u}_A{A}_E+\left(1-{u}_A\right)\left({B}_P+{B}_M\right)\right\}. $$

Because the right-hand side of Eq. (13) is always negative, *A*_*M*_ continuously decreases. The population of the mutualist strain therefore never increases for any parameter values under unlimited dispersal conditions. This result also applies to Sp. B. Thus, mutualism cannot evolve in either species in this system.

## Limited dispersal

Now, consider the case for limited dispersal. I employed a simulation to investigate the ability of the mutant strains to invade populations of the “wild-type” strains, using Monte-Carlo simulations following the procedure described by Yamamura et al. (2004). I constructed the dual lattice in a 25 × 25 torus space, starting with wild-type individuals of both species on all sites on both lattices, and ran the model for 1,000 generations to attain a state of quasi-equilibrium. In each generation, the following events occur. (1) A site is chosen at random on lattice A. (2) If there is an individual of Sp. A present, it produces offspring which occupy any empty neighbouring sites (Neumann neighbourhood), with a probability *R*_*A*_/4 for each empty neighbouring site, where *R*_*A*_ is the reproductive rate of the parent individual. This rate depends on the status of the corresponding site on lattice B. (3) If there is an individual of Sp. B present at the corresponding site on lattice B, the same process is applied to this individual. (4) The Sp. A individual is killed with probability *D*_*A*_, which depends on the status of the corresponding site on lattice B. (5) The same procedure is conducted for the corresponding Sp. B individual. (6) The above process is repeated 25 × 25 times. Mutant individuals are then introduced at random, irrespective of whether the site is occupied or not, at a frequency of 0.04 (25 individuals on one lattice if the partner species does not change its behaviour or on each lattice if mutant strains are introduced for both species). If a wild-type individual is present, it is replaced by the mutant individual. After running this simulation for 1,000 generations, the frequency of mutant individuals in the wild-type population is noted. I ran the above procedure 100 times to derive the ensemble mean of the resulting frequencies. The invasiveness of the mutualistic strain was examined by recording the frequency with which its population increased after the introduction. The invasiveness of mutant parasitic individuals into a wild-type mutualistic population (hereafter, reciprocal invasiveness) is also examined to check the robustness of mutualism.

In this study, I focus on evolutionary conditions that cause differential influences of interspecific interaction on the two fitness components: fecundity and survival. For simplicity, I therefore assume symmetry between the quantities of resources that are lost and gained by each species, i.e., *b*_*X*_ = *b′*_*X*_ and *c*_*X*_ = *c′*_*X*_ (*X*: *A*, *B*), and ran the simulation as coded above, using several values for *c*_*X*_ and *b*_*X*_. I restricted the parameters *p*_*X*_, *q*_*X*_, *u*_*X*_, and *v*_*X*_ to either zero or one, because the number of possible combinations of intermediate values between zero and one is unfeasibly large. I also assume symmetry between the effects of parasitism and mutualism, i.e., *c*_*A*_ = *b*_*B*_ and *c*_*B*_ = *b*_*A*_, and between the reproductive and mortality rates of each species, i.e., *r*_*A*_ = *r*_*B*_ = 0.5 and *d*_*A*_ = *d*_*B*_ = 0.2.

## Results

**Fig. 2:**
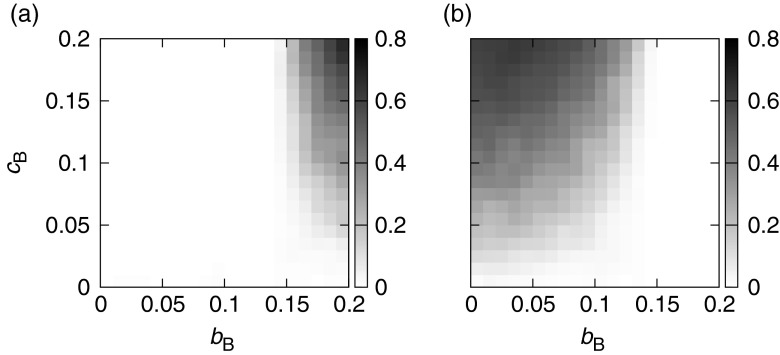
The invasiveness of the mutualistic strain and the robustness of the mutualist strain to invasion by the parasitic strain in a host-symbiont system. Sp. A is the host species, and the cost and benefit to it are fixed as 0 (*c*_A_ = *b*_A_ = 0.0). The horizontal axis represents the benefit to the symbiont (Sp. B) exploiting resources taken from Sp. A, and the vertical axis represents the cost to the mutualistic strain of Sp. B (Sp. BM) contributing to Sp. A. Panel **a** shows the vulnerability of a population of the parasitic strain of Sp. B (Sp. BP) to invasion by Sp. BM, i.e., the ensemble-mean frequency of Sp. BM. The result of the reverse process, i.e., the ensemble-mean frequency of Sp. BP after it invaded into a Sp. BM population, is illustrated in **b**

First, assume that Sp. A does not affect Sp. B at all, i.e., *c*_*A*_ = *b*_*A*_ = 0 (in this case, parameters *p*_*A*_, *q*_*A*_, *u*_*A*_, and *v*_*A*_ are meaningless), whereas Sp. B behaves as either a parasite or a mutualist with respect to Sp. A, depending on the values of *p*_*B*_, *q*_*B*_, *u*_*B*_, and *v*_*B*_. I examined the ability of a mutualistic strain (hereafter, Sp. BM), described by *p*_*B*_, *q*_*B*_, *u*_*B*_, and *v*_*B*_ (*c*_*B*_ ≠ 0, *b*_*B*_ ≠ 0), to invade a population of the parasitic strain (Sp. BP), described by *p*_*B*_ and *q*_*B*_ (*c*_*B*_ = 0, *b*_*B*_ ≠ 0), under the condition that the behaviour of their neutral host, Sp. A, is fixed as *c*_*A*_ = *b*_*A*_ = 0. In most cases, the mutualistic strain failed to invade into the parasite population. However, when (*p*_*B*_, *q*_*B*_) = (0, 1) for both strains and (*u*_*B*_, *v*_*B*_) = (1, 0) for the mutualistic strain, the results were more interesting. In this scenario, Sp. BP used resources affecting the fecundity of Sp. A for its survival, but Sp. BM increased the survival of Sp. A by reducing its own fecundity. I conducted a test of reciprocal invasiveness under this scenario, i.e., the invasion of Sp. BP into a Sp. BM population. Figure 2a illustrates the ensemble-mean frequency of Sp. BM (*B*_*M*_) 1,000 generations after the generation in which it was introduced into a population of Sp. BP. The mutualists achieved higher frequency when both the exploitation by Sp. B (*b*_*B*_) and the contribution made by Sp. BM (*c*_*B*_) were high. Figure 2b illustrates the results of the reverse process, with an introduction of Sp. BP into a population of Sp. BM. Comparing Fig. 2a, b, we find that under conditions in which one strain can increase in the population of the other, the reverse process is impossible. The invasiveness of the mutualistic strain under several parameter settings was examined (Online resources). The parameter region in which the population is vulnerable to invasion shrinks along the y-axis if *c′*_*B*_ > *c*_*B*_ (Online resource 1) and widens along the x-axis if *b′*_*B*_ > *b*_*B*_ (Online resource 2). This means that low levels of both exploitation and altruism cannot lead to the establishment of mutualism. The patterns of invasiveness are the same under a range of background reproductive and mortality rates (data not shown).

**Fig. 3:**
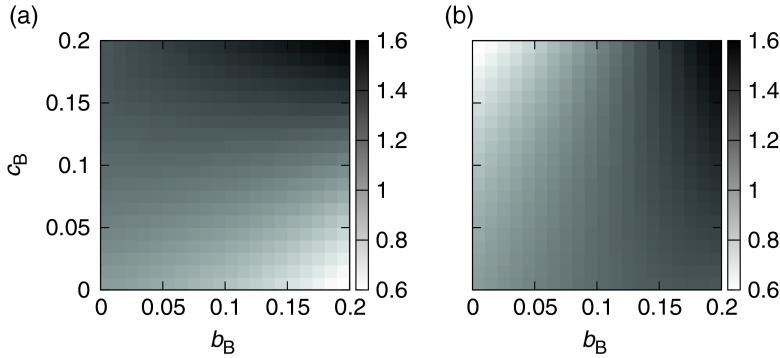
The sensitivity of population size to the cost and/or benefit to the species of the host-symbiont system (*c*_A_ = *b*_A_ = 0.0). The graphs show host (**a**) and symbiont (**b**) population size relative to the situation in which there is no interaction between the host and symbiont (*c*_B_ = *b*_B_ = 0.0)

It is still unclear whether the mutant mutualistic strain really plays a mutualistic role for its host species even if its population increases, because all symbiotic strains essentially play a parasitic role in this model. The population size of the host tells us whether the mutualistic strain does or does not affect it positively. In dual lattice models used in previous studies, the mutualist succeeds in invasion and increases its population only if the mutualists interact with each other at the same position (Yamamura et al. 2004). In the present model setting, there is a possibility that the mutualistic strain nevertheless decreases the host population, meaning the mutualistic strain actually plays a parasitic role. The ratio of the ensemble-mean population of the host species interacting with a mutualistic strain to that of the free-living host species at equilibrium, i.e., in the absence of invasion, was checked in this regard. If the ratio falls below 1.0, then the symbiont, whether it behaves as a parasite or a mutualist, plays an overall parasitic role. Figure 3a shows the ratio between the long-term (1,000 generations) average population size of Sp. A interacting with Sp. BM and population size without Sp. B (i.e., *c*_B_ = *b*_B_ = 0). On the horizontal axis, the situation is identical to Sp. A interacting with Sp. BP, because Sp. BM does not incur any cost for its mutualistic behaviour (i.e., *c*_B_ = 0). In fact, Sp. A increases its population when Sp. BM is strongly exploitative and also pays a large cost, whereas Sp. A’s population decreases when the mutualistic strain is strongly exploitative and pays a small cost. Figure 3b illustrates the increase and/or decrease in population size of Sp. BM in a similar way to Fig. 3a. The population of Sp. BM increases when both *b*_*B*_ and *c*_*B*_ are high, as does that of its host, Sp. A. Paying a cost greater than the benefit derived from exploiting Sp. A results in a decrease in the population of Sp. BM. This leads to an increase in the population of Sp. BP after it invades the mutualist population (Fig. 2b). It is notable that Sp. A’s population keeps increasing as the exploitation rate increases, regardless of the reduction in its fecundity, showing that Sp. A is able to reproduce well in association with Sp. BM. The reduction in mortality caused by this association is therefore more advantageous to the population than an increase in fecundity.

**Fig. 4:**
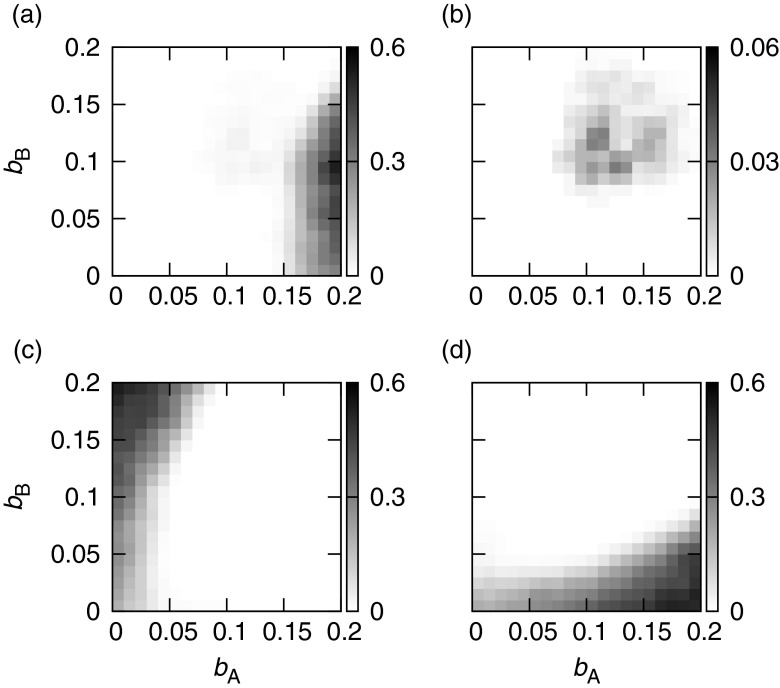
Test of the invasiveness of both mutant mutualistic strains into wild-type parasitic populations and vice versa. Panels **a** and **b** are the result of mutualistic strain invasion into parasitic populations, and panels **c** and **d** are the result of the reciprocal invasiveness test, in relation to the cost/benefit parameters on each lattice. Each panel shows the ensemble-mean frequency resulting from the combination of different strains of Sp. A and Sp. B. Panel **a** represents the mutualistic strain of Sp. A (Sp. AM) interacting with the parasitic strain of Sp. B (Sp. BP); **b** represents Sp. AM interacting with Sp. BM; **c** indicates Sp. AP regardless of its interacting partner; and **d** indicates Sp. BP regardless of its interacting partner. The horizontal axis represents the benefit to Sp. A of exploiting Sp. B, and the vertical axis represents the benefit to Sp. B. The cost of mutualistic behaviour for both species is fixed in all cases (*c*_A_ = *c*_B_ = 0.1)

I then simulated the case where both species behave as parasites at the beginning and mutualists invade on both lattices. For simplicity, model parameters were symmetrical between species, i.e., (*p*_A_ = *p*_B_), (*q*_A_ = *q*_B_), (*u*_A_ = *u*_B_), and (*v*_A_ = *v*_B_). In this simulation, the mutualistic strains each pay the same cost (*c*_*A*_ = *c*_*B*_ = 0.1). Under these conditions, the mutualistic strains never increase their populations after invasion except when (*p*_A_, *q*_A_) = (*p*_B_, *q*_B_) = (0, 1) and (*u*_A_, *v*_A_) = (*u*_B_, *v*_B_) = (1, 0). Here, I define success in the establishment of mutualism as an increase in the population of the mutualistic strain, i.e., the ensemble-mean frequency of each mutualistic strain should be >0.04 after the invasion. Figure 4a, b shows the capabilities of the mutaulistic strains of both species to invade into their parasitic populations, and Fig. 4c, d illustrates the result of the reverse invasion test. Cells on each lattice can have one of nine different states as follows: (A_i_, B_j_), where (i, j ∈ P, M, E). The ensemble-mean frequency that results when Sp. AM interacts with Sp. BP is shown in Fig. 4a and that of Sp. AM interacting with Sp. BM in Fig. 4b. The result for Sp. BM is the same as that shown in Fig. 4a, b, but transposed x-axis to y-axis, as the model parameters for Sp. A and Sp. B are symmetrical in this case. Patch occupancy at the same position in both lattices by the mutualistic strains of both species is unusual (Fig. 4b), suggesting that the establishment of mutualism in both species at the same time is unlikely. Indeed, a mutualistic strain can invade the population of a parasitic strain only if its partner on the other lattice is parasitic (Fig. 4a). This situation is essentially identical to the host-symbiont system shown in Fig. 2. The population of the parasitic strain increases as a result of interacting with the mutualistic host, because of the survival advantage; the mutualistic host is then able to outcompete the parasitic strain on the same lattice, and its population increases in turn. However, the population of the mutualistic strain does not increase if the exploitation rate is low. The threshold parasitic exploitation rate that allows the establishment of mutualism is *b*_A_ = 0.15 (Fig. 4a). In this case, the improved survival rate confers a greater advantage than a corresponding increase in fecundity. Figure 4c, d illustrates the results of the reverse invasion test. The ensemble-mean frequency of Sp. AP (regardless of its interacting partner) is shown in Fig. 4c and that of Sp. BP in Fig. 4d. The region where Sp. AP is invasive (Fig. 4c) never overlaps with that in which Sp. AM is capable of invading parasite populations (Fig. 4a, b), but that for Sp. BP (Fig. 4d) overlaps partially. These results suggest that a stable association between Sp. AM and Sp. BM is unlikely to occur, but that associations between Sp. AM and Sp. BP or between SP. AP and Sp. BM are stable. When the exploitation rates of the two species become large enough (*b*_A_ > 0.1 and *b*_B_ > 0.1), the parasitic strains never successfully invade mutualistic populations. A mutually mutualistic relationship between the two species will therefore always be robust, although such an association can only become established infrequently. This scenario is unrealistic because the mortality rates of both mutualists fall below 0.0 if the exploitation rate is higher than 0.1.

## Discussion

I have shown that a high level of parasitic exploitation and a large cost of altruistic behaviour are both required for mutualism to evolve from parasitism and become established in a host-symbiont system (Fig. 2). In other words, mutualism never becomes established if a parasite has a low exploitation rate. This result is totally different from the evolutionary scenario in which decreasing virulence is required for the establishment of mutualism from parasitism (Ewald 1987) and suggests a new narrative of the evolution of mutualism, in which mutualism can become established without this condition. This new condition for the establishment of mutualism is always true, even if the values of the resources exchanged between host and symbiont are not equal, i.e., *c*_*B*_ ≠ c′_*B*_ or *b*_*B*_ ≠ *b*′_*B*_ (Online resources). This novel finding arises from the separation of the fitness components in this model, as opposed to previous studies in which the costs and benefits of interactions were included in the same fitness component (Doebeli and Knowlton 1998; Yamamura et al. 2004). It is natural that a conflict between the host and symbiont in terms of virulence arises, and the shift of transmission mode or the utilisation of waste product resolves this conflict in previous studies (Yamamura 1993, 1996; Genkai-Kato and Yamamura 1999). In the present results, the conflict in virulence (parasitic benefit in this model) between the host and parasite is reflected in the decreases and increases in population size on the horizontal axis in Fig. 3a, b. Natural selection would tend to reduce parasitic exploitation if the cost-free positive effect of the mutualistic symbiont on the host species (Genkai-Kato and Yamamura 1999) led to an increase in the host population, which in turn would lead to an increase in encounter rate between the host and mutualist. However, the existence of an altruistic cost to the mutualist drives natural selection in the opposite direction. Consequently, both populations increase at high exploitation rates. The invasion of a mutualist strain into a parasitic population and the reverse process are not possible if the parasite has an intermediate exploitation rate and the mutualist pays a small cost for its altruism (Fig. 2). This suggests that a decrease in virulence is not the evolutionary path for the establishment of mutualism.

Another important factor to consider is vertical transmission (Ewald 1987). In fact, the cluster structure used here ensures the transmission of the symbiotic partner to the next generation for both the host and the symbiont species. On the other hand, however, a closely packed population structure deprives offspring of the opportunity to settle in a vacant patch close to their parents. In such a situation, a high reproductive rate confers very little advantage compared to a low mortality rate. Furthermore, a positive effect on the fecundity of the host should cause the fragmentation of the host-mutualist cluster, because the abundant newborn individuals of the host species in the surrounding patches create opportunities for the parasitic strain to interact with neutral host individuals.

The simultaneous establishment of mutualism in two different parasitic species never occurred in this study (Fig. 4b), although previous studies have shown that the establishment of mutualist strains of two species can occur in a coordinated manner (Yamamura et al. 2004). Doebeli and Knowlton (1998), who originally developed the dual lattice model, shed light on the nature of cooperation between two species, and the study by Yamamura et al. (2004), which built on Doebeli and Knowlton’s work, agrees with other work on the spatially structured IPD game. Their model incorporates costs and benefits to each species but ignores the negative effect on one species caused by the parasitic behaviour of the other (Doebeli and Knowlton 1998; Yamamura et al. 2004; Travis et al. 2006). In this scenario, when resource exchange between symbiotic partners was modelled, mutualism that developed from parasitism never became established, but if established was very resistant to invasion. However, mutualists were allowed to develop only if it interacts with its parasite (Fig. 4a). This result strengthens our view that mutualism evolves from parasitism (Roughgarden 1975; Ishikawa 1988), because it suggests that one of the altruistic behaviours of a mutualistic unit consisting of two species must originate as a parasitic behaviour.

Evolution of symbiosis via resource exchange has been investigated theoretically (Law and Dieckmann 1998; Ferriere et al. 2002). Especially, Law and Dieckmann (1998) focused on the emergence of symbiotic unit consisted of two genetically distinct lineages from free-living state. In their model, both species exploit resources from each other, i.e., behaves parasitically, which situation is similar to this study. An increase in the fecundity of symbiotic unit as symbiotic benefit and an increase mortality of free-living as cost are assumed in their model from the experimental study on the evolution of a bacterial infection of *Amoeba* (Jeon 1972), and it is highlighted that the mortality of free-living is higher than that of symbiotic unit, which consists with this study. However, the result of this study demonstrates that the bacteria-*Amoeba* symbiosis, which they modelled on, must have a benefit to its survival and a cost to its fecundity to exist.

This study demonstrates the importance of the exact mechanism in which a cost/benefit affects fitness in determining the nature of a close relationship between two species. There are examples of symbiotic species pairs in which the symbiont decreases not only the mortality of the host species but also negatively affects its reproduction. The secondary endosymbiont of the aphid, *S. symbiotica* (Koga et al. 2003), and *Wolbachia* in *D. melanogaster* (Brownlie et al. 2009) are examples of symbionts that provide beneficial metabolites to their host species, even though they are in essence parasites. Cases of symbiont-mediated host protection (Jones et al. 2007; Haine 2008), for example *Wolbachia* suppressing an anti-RNA virus in *D. melanogaster* (Hedges et al. 2008; Teixeira et al. 2008), are also examples of the type of association modelled in this study. In these cases, the symbiont produces anti-microbial peptides and thereby provides a benefit to its host, even though the symbiont functions as a parasite when the host is healthy. These examples include only vertically transmitted symbionts, but this model can be expanded to include cases of imperfect vertical transmission, such as those that are seen in plant and fungi symbiosis. Endophytes are not always transmitted vertically to their host plants, and the dispersal of their offspring and that of their host plant is narrow (Saikkonen et al. 1998). This is the exact situation assumed in the model here. In this case, the mutual relationship between the host and symbiont would be maintained by the symbiont improving its host’s immunity, as has been shown empirically (Clay 1988; Faeth and Sullivan 2003). The endophyte does this by producing alkaloid chemicals with anti-insect effects. These instances are examples of associations in which the symbiont has a benefit to the host in that it decreases its mortality. The costs and benefits to the symbiont of providing this benefit to the host (i.e., decrease in fecundity and increase in survival) have not yet been clearly demonstrated. However, it is highly likely that producing amino acids or peptides for use by the host reduces the symbiont’s growth rate because it must consume the symbiont’s resources. The symbiont therefore decreases its fecundity (experiences a cost) in order to provide a benefit to its host species.

The trigger that leads to the establishment of mutualism in this model, and in some actual cases listed above, is the benefitial effect of the symbiotic partner on the survival of the host. It is likely that in nature, changes in environmental conditions such as nutritional shortages or epidemics directly affect the survival of the host species and thereby facilitate the establishment of mutualism. In the model framework, however, the close-packed population structure actualises this effect on mortality. We can consider that the mutualistic symbiont provides a new function of resource allocation to the host species, in which resources are shifted from fecundity to survival (Fellous and Salvaudon 2009). In fact, the metabolites extracted from or provided by its symbiotic partner are not used for only enhancing either fecundity or survival, which corresponds to intermediate values of the parameters *p*, *q*, *u*, and *v*. However, the positive effect on survival resulting from the resource exchange between symbiotic partners might nevertheless be sufficient to allow the establishment of mutualism. Models estimating geometric mean fitness in a fluctuating environment emphasise survival (Yoshimura and Jansen 1996; Yoshimura 2009). Fellous and Salvaudon (2009) suggested that the evaluation of mutualistic symbiosis involving both positive and negative effects should be based on the geometric mean fitness of the host species, but they did not consider the question of the origin of mutualism. It is therefore plausible to assess the evolution and maintenance of parasitic mutualism using geometric mean fitness.

## Electronic supplementary material

Below is the link to the electronic supplementary material.

Online resource 1 (DOCX 354 kb)

Online resource 2 (DOCX 347 kb)
