# Supplementary material for: Evolution of symbiosis with resource allocation from fecundity to survival
Source: Naturwissenschaften. 2014 Apr 18;101(5):437–46. doi: 10.1007/s00114-014-1175-1 (PMC4012156; doi:10.1007/s00114-014-1175-1)
Supplement: Supplementary file 2 — Online resource 2 (DOCX 347 kb) [file 114_2014_1175_MOESM2_ESM.docx]

The invasiveness of the mutualistic strain and the robustness of the mutualist strain to invasion by the parasitic strain in a host-symbiont system when *bʹ_B_* > *b_B_*. Sp. A is the host species, and the cost and benefit to it are fixed as 0 (*c*_A_ = *b*_A_ = 0.0). The horizontal axis represents the benefit to the symbiont (Sp. B) exploiting resources taken from Sp. A, and the vertical axis represents the cost to the mutualistic strain of Sp. B (Sp. BM) contributing to Sp. A. Panel (a) shows the capability of Sp. BM to invade into a population of the parasitic strain of Sp. B (Sp. BP), i.e. the ensemble-mean frequency of Sp. BM. The result of the reverse process, i.e. the ensemble-mean frequency of Sp. BP after it invaded into a Sp. BM population, is illustrated in (b). The parameters of resource allocation are (*p_B_*, *q_B_*, *u_B_*, *v_B_*) = (0, 1, 1, 0) just the same as those shown in Figure 2 except for the condition (*b_B_* = 2 *bʹ_B_*). Reproductive and mortality rates are fixed as *r_A_* = *r_B_* = 0.5, and *d_A_* = *d_B_* = 0.2. This situation means the exploited material from Sp. A is worth for Sp. B more than Sp. A. The parameter region of invasiveness expands along with x-axis as compared with that shown in Figure 2. However, the mutualism establishment is never allowed if both the parasitic exploitation by Sp. B (*b_B_*) and the contribution made by Sp. BM (*c_B_*) were low levels, which pattern is just the same as that shown in Figure 2.
